# Predictive modeling and socioeconomic determinants of diarrhea in children under five in the Amhara Region, Ethiopia

**DOI:** 10.3389/fpubh.2024.1366496

**Published:** 2024-08-01

**Authors:** Abdulaziz Kebede Kassaw, Ayana Alebachew Muluneh, Ebrahim Msaye Assefa, Ali Yimer

**Affiliations:** ^1^Department of Health Informatics, College of Medicine and Health Sciences, Wollo University, Dessie, Ethiopia; ^2^Department of Pre-clerkship, College of Medicine and Health Science, Wollo University, Dessie, Ethiopia; ^3^Department of Public Health, College of Health Sciences, Woldia University, Woldia, Ethiopia

**Keywords:** machine-learning, diarrhea, under five children, prediction modeling, Ethiopia

## Abstract

**Background:**

Diarrheal disease, characterized by high morbidity and mortality rates, continues to be a serious public health concern, especially in developing nations such as Ethiopia. The significant burden it imposes on these countries underscores the importance of identifying predictors of diarrhea. The use of machine learning techniques to identify significant predictors of diarrhea in children under the age of 5 in Ethiopia’s Amhara Region is not well documented. Therefore, this study aimed to clarify these issues.

**Methods:**

This study’s data have been extracted from the Ethiopian Population and Health Survey. We have applied machine learning ensemble classifier models such as random forests, logistic regression, K-nearest neighbors, decision trees, support vector machines, gradient boosting, and naive Bayes models to predict the determinants of diarrhea in children under the age of 5 in Ethiopia. Finally, Shapley Additive exPlanation (SHAP) value analysis was performed to predict diarrhea.

**Result:**

Among the seven models used, the random forest algorithm showed the highest accuracy in predicting diarrheal disease with an accuracy rate of 81.03% and an area under the curve of 86.50%. The following factors were investigated: families who had richest wealth status (log odd of −0.04), children without a history of Acute Respiratory Infections (ARIs) (log odd of −0.08), mothers who did not have a job (log odd of −0.04), children aged between 23 and 36 months (log odd of −0.03), mothers with higher education (log odds ratio of −0.03), urban dwellers (log odd of −0.01), families using electricity as cooking material (log odd of −0.12), children under 5 years of age living in the Amhara region of Ethiopia who did not show signs of wasting, children under 5 years of age who had not taken medications for intestinal parasites unlike their peers and who showed a significant association with diarrheal disease.

**Conclusion:**

We recommend implementing programs to reduce the incidence of diarrhea in children under the age of 5 in the Amhara region. These programs should focus on removing socioeconomic barriers that impede mothers’ access to wealth, a favorable work environment, cooking fuel, education, and healthcare for their children.

## Introduction

The World Health Organization (WHO) defines diarrhea as passing at least three loose stools in a day, usually due to an intestinal tract infection caused by bacteria, viruses, or parasites. Bacterial causes include Salmonella, Shigella species, *Escherichia coli*, *Campylobacter jejuni*, and *Vibrio cholera*. Parasitic causes include Giardia, Entamoeba, Cryptosporidium, and helminths, while viral causes include rotavirus and adenovirus (1).

Diarrhea is a major public health concern, reported as the second leading cause of death among children under the age of 5 (2, 3). Globally, 1 in 9 children under the age of 5 died due to diarrhea (2). It accounts for killing approximately 525,000 children and 1.7 billion cases every year among children under the age of 5, with the highest number reported in sub-Saharan Africa (3, 4). According to the 2016 Ethiopian Demographic and Health Survey (EDHS) report, the prevalence of diarrhea among children under the age of 5 within 2 weeks prior to the survey was 12% (5).

According to the Integrated Global Plan of Action for the Prevention and Control of Pneumonia and Diarrhea (GAPPD), there is an approach to ending mortality caused by pneumonia and diarrhea by 2025 that encompasses both vital services and interventions to create a healthy environment; it inspires practices that guard children against disease and provides access to recognized and appropriate prevention and treatment measures. Therefore, the approach has aimed to decrease diarrhea mortality in children under the age of 5 to less than 1 per 1,000 live births (6).

Diarrhea is a global problem, particularly in developing countries, as it accounts for the majority of deaths in children under the age of 5. Since diarrhea needs to be treated urgently and timely to minimize complications, it is better to identify the crucial factors for decisive treatment and better results. Therefore, effective and integrated intervention mechanisms that leverage scientific research are critical to addressing this deadly and devastating public health problem, and studies on the predictors of diarrhea in children under the age of 5 are of paramount importance. Children with diarrhea face numerous problems, such as loss of appetite and inadequate nutrient intake, which can potentially lead to weight loss and stunted growth. Diarrhea also causes water and electrolyte deficiencies if not replaced in a timely manner, and dehydration is the fatal complication of diarrhea (7). Several studies conducted in the past have found that factors such as socio-demographic, maternal, environmental, and nutritional factors are some of the determinants of diarrhea in children under the age of 5 (4, 8–11).

Diarrheal disease remains a public problem, although preventative measures have been taken. Previous studies have provided much evidence on the socioeconomic and demographic factors significantly associated with diarrhea in children under the age of 5 in Ethiopia (4, 11, 12). They focused on classical methods or using traditional regression models to determine risk factors associated with diarrheal disease. In this study, we predicted the important determinants of diarrhea among children under the age of 5 in Ethiopia using non-classical regression models extracted from regionally and nationally representative data.

Currently, the healthcare sector produces huge amounts of data about patients and disease diagnoses, and when these data are well processed and analyzed using robust methods, they provide important knowledge that can be used competently in decision-making, healthcare management, disease detection, and diagnosis. Therefore, this sparked the researcher’s interest in using a machine learning approach to predict determinants of diarrhea in children under the age of 5 in Ethiopia’s Amhara regional state.

This study enables the government and other stakeholders to gain deep insights into risk factors and clearly identify where to direct resources for improved prevention with early intervention with proven effectiveness. It also highlights the importance of a machine learning-based approach to diarrheal disease prediction and will help data scientists and other scientists in further research.

## Methods

### Data source

**Figure 1:**
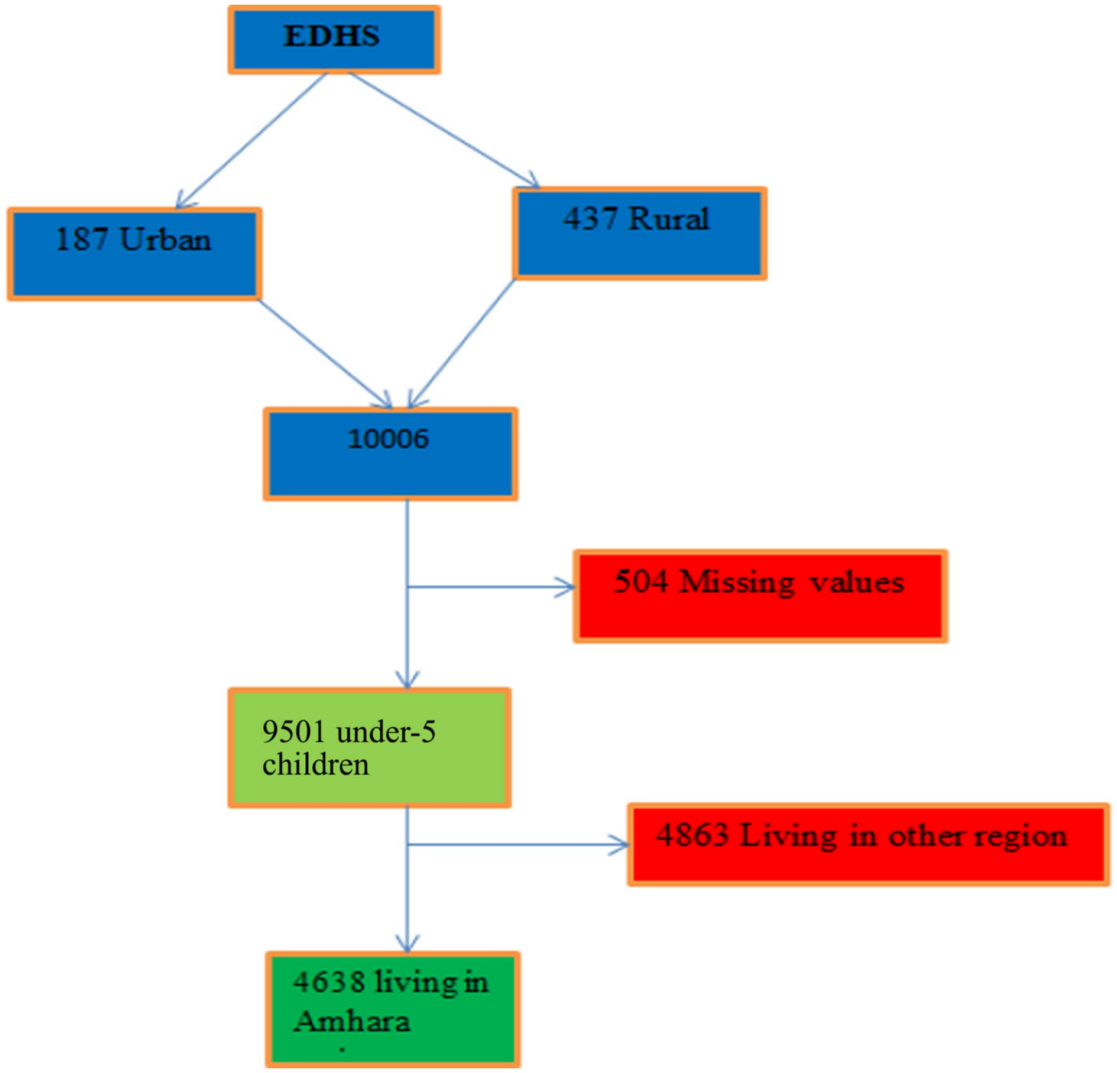
Sampling procedure for study dataset.

The EDHS provided the study’s data. Ethiopia took part in the EDHS for the fourth time as a participant in the global demographic and health surveys program. The study was carried out using a cross-sectional study design and was conducted from 18 January to 27 June 2016. A multi-stage stratified sampling technique based on Ethiopia’s 2007 national population and housing census was used in this nationally representative household survey to select respondents from a total of 624 clusters spread throughout nine regions and two administrative cities (187 urban and 437 rural). Children under the age of 5 make up the unit of analysis, and a total of 10,006 children were chosen from 624 clusters throughout Ethiopia for the sample. All reproductive women who had at least one child under the age of 5 prior to the survey participated. There were 9,501 children in Ethiopia under the age of 5. Out of 9,501, 4,638 under-5 residents of Amhara Regional State made up the study’s sample size after missing data were eliminated in this study (Figure 1).

#### Inclusion and exclusion criteria

Those children under the age of 5 who had complete data on the 2016 EDHS data set were included in the current study, but participants with insufficient data and missing values were excluded from this study.

### Study variables and measurements

This study considered the children’s recode (KR) file from the 2016 EDHS dataset. The outcome variable is “a child had diarrhea or not prior to two weeks of the survey,” which was measured as a binary outcome as absence of diarrhea (coded as zero) or presence of diarrhea (coded as one) for all the models.

Based on the dataset’s accessibility and the known theoretical relationships from the literature, we selected certain variables for our investigation as coded in the EDHS (13–17). Some variables were created by recording values of separate variables or combining two or more variables. The following factors were selected as probable diarrhea predictors: the child’s age (less than 6 months, 6–11, 12–23, 24–35, 36–47, and 48–59 months); the child’s place of residence (rural or urban); the mother’s educational level (primary, secondary, and above); the cooking fuel (wood, charcoal, or electricity); the wealth quintile (poorest, poor, middle, rich, and richest); maternal employment status; and the child’s sex (male or female). Other factors included breastfeeding status (never, ever, not currently); vitamin A supplementation (yes, no); recent acute respiratory infection (ARI) (no, yes); the number of children still alive (1–3, 4–6, above 6); and drinking water sources (labeled as either “improved” or “unimproved”) (18). The evaluation of children’s nutritional status involved the computation of z-scores for “height-for-age (stunting)” and “weight-for-height (wasting)” using child physical growth indicators recommended by WHO (13, 19). Children were classified as stunted or wasted if their z-score for each nutritional status was two standard deviations lower than the WHO reference population median (13, 19) and media exposure was defined as “yes,” meaning the child had access to at least one form of media (radio, TV, or newspaper); “no” meant they had none. These predictor variables were chosen based on previously published works on the subject (4, 12).

### Data analysis

Data analysis for this study was conducted in two steps. In the first stage, data relevance analysis and descriptive data visualization were completed using statistical tools (R software). The data were converted to comma-delimited (CSV) format. The second stage involved preprocessing the data using RStudio and Python with an Anaconda notebook, including data cleaning and handling missing values (13).

#### Feature selection methods

Feature selection and variable importance rank (20, 21) were techniques for identifying a subset of features by removing irrelevant or redundant features. The significance of feature selection lies in reducing the cost of learning by limiting the number of features. The Boruta algorithm was chosen for feature selection in this investigation. The Boruta algorithm infers the relevance of features based on the random forest estimate of their importance and identifies both highly and weakly relevant features from the dataset (22).

#### Data split

Data splitting (23) involves separating the data into two sets: an explicit training dataset to build the model and an unseen test dataset to assess the model’s performance on new data, applying an 80:20 ratio.

#### Imbalanced data handling

As its name suggests, imbalanced data (24) indicate when the data proportion in the outcome variable is disproportionate. If the prediction contains and imbalanced data set, it will affect the result. So imbalanced data handling is a way of avoiding biased prediction results. As a result we applied all imbalance data handling methods like the Under, Over, Smote, Rose and ensemble balancing method and selected the SMOTE one from those depending based on performance.

#### Building a predictive modeling

Predictive modeling builds a statistical model of future behavior using the trained dataset as a basis. In machine learning, predictive modeling uses a set of predictor variables to forecast an outcome’s likelihood (25). Depending on whether the dependent variable is a binary response (yes/no), different machine learning algorithms for classification can be applied (26). This study used machine learning prediction methods, including logistic regression, gradient boosting, random forest, naïve Bayes classifier, decision tree (C5.0), and support vector machine with three distinct kernels (27). A balanced dataset was used for every prediction algorithm to improve prediction skills.

#### Performance evaluation for predictive models

The performance of the prediction models was assessed using several common evaluation criteria, including ROC curve, accuracy with confusion matrix, and Kappa statistics (28) which is represented as;

**Table:** 

*N*=Number of instances		Confirmed by observation	
Predicted by test		Yes	No
	Yes	TP (Presence of disease)	FP (Type 1 error)
	No	FN (Type 2 error)	TN (absence of disease)
TP, true positive; FP, false positive (type I error); FN, false negative (type II error); TN, true negative.			

True positive rate (TPR), false positive rate (FPR), precision, and recall can be calculated as mentioned in Eqs. (1−7).


(1) True positive rate(TPR)=TP/(TP+FN).

(2) $$\mathrm{Precision}\;\left(\mathrm{positive}\;\mathrm{predictive}\;\mathrm{value}\right)=TP/\left(TP+FP\right)\text{.}$$

(3) $$\mathrm{Negative}\;\mathrm{predictive}\;\mathrm{value}=TN/\left(TN+FN\right)\text{.}$$

(4) $$\mathrm{Specificity}\;\left(\mathrm{true}\;\mathrm{negative}\;\mathrm{rate}\right)=TN/\left(TN/FP\right)\text{.}$$

(5) ROCisatrade−offcurvedrawnbetweenTPRandFPR.

(6) $$\mathrm{Accuracy}\;\left(\%\right)=\left(\left(TP+TN\right)\backslash \left(TP+TN+FP+FN\right)\right)\times 100.$$

(7) $$\mathrm{Balanced}\;\mathrm{accuracy}=1/2\left(\mathrm{sensitivity}+\mathrm{specificity}\right)\text{.}$$

### Unsupervised machine learning for diarrhea prediction

Unsupervised machine learning analyzes input data to identify important structures or patterns not immediately apparent. In this machine learning experiment, the model was neither trained nor monitored by users. It finds previously undetected patterns and information over time (28).

### Association rules

An unsupervised prediction rule association was used in this analysis section. Several rules for classification or prediction were produced by the rule-based prediction method, with significant rules selected based on performance measurement criteria. Important guidelines were chosen using the lift (14, 15), an interesting quality assessment criterion for the association. Lift measures the positive or negative correlation between the antecedent (if) and consequent (then) of a rule. It is calculated as the ratio of the rule’s confidence to the likelihood that the consequence will occur. It is defined as the ratio of the dependent variable’s (B) occurrence probability to the independent variable’s (A) condition (Eq. 8):


(8) $$\mathrm{Lift}\;\left(A\to B\right)=\frac{c\left(A\to B\right)}{P\left(B\right)}=\frac{P\left(A\right)}{P\left(A\right)P\left(B\right)}$$

The lift value range is [0, +∞). If lift equals 1, it indicates that X and Y occurring simultaneously are independent random events with no particular meaning, suggesting no correlation between A and B. These are known as uncorrelated rules. If the lift value is less than 1, indicating that the occurrence of “A” reduces the occurrence of “B,” these are referred to as negative correlation rules. If the lift value is greater than 1, indicating that the occurrence of “A” encourages the occurrence of “B,” these are referred to as positive correlation rules.

### Hyperparameter tuning

A working model parameter is an external characteristic of the model whose value is user-specified because it cannot be understood from the data (16). The Optuna framework was used to tune hyperparameters for this study (17). To better understand the likelihood of the optimal values avoiding unnecessary estimation for the combination of underperforming parameters in the search for the ideal parameter settings, the authors explain how Optuna operates. Specifically, they describe hyperparameter optimization as a process of minimizing or maximizing an objective function that takes a set of hyperparameters as an input (17). This approach works better than traditional hyperparameter tuning techniques such as grid search and randomized search, which effectively maximize the model using the user’s provided hyperparameters.

### Making predictions

**Figure 2:**
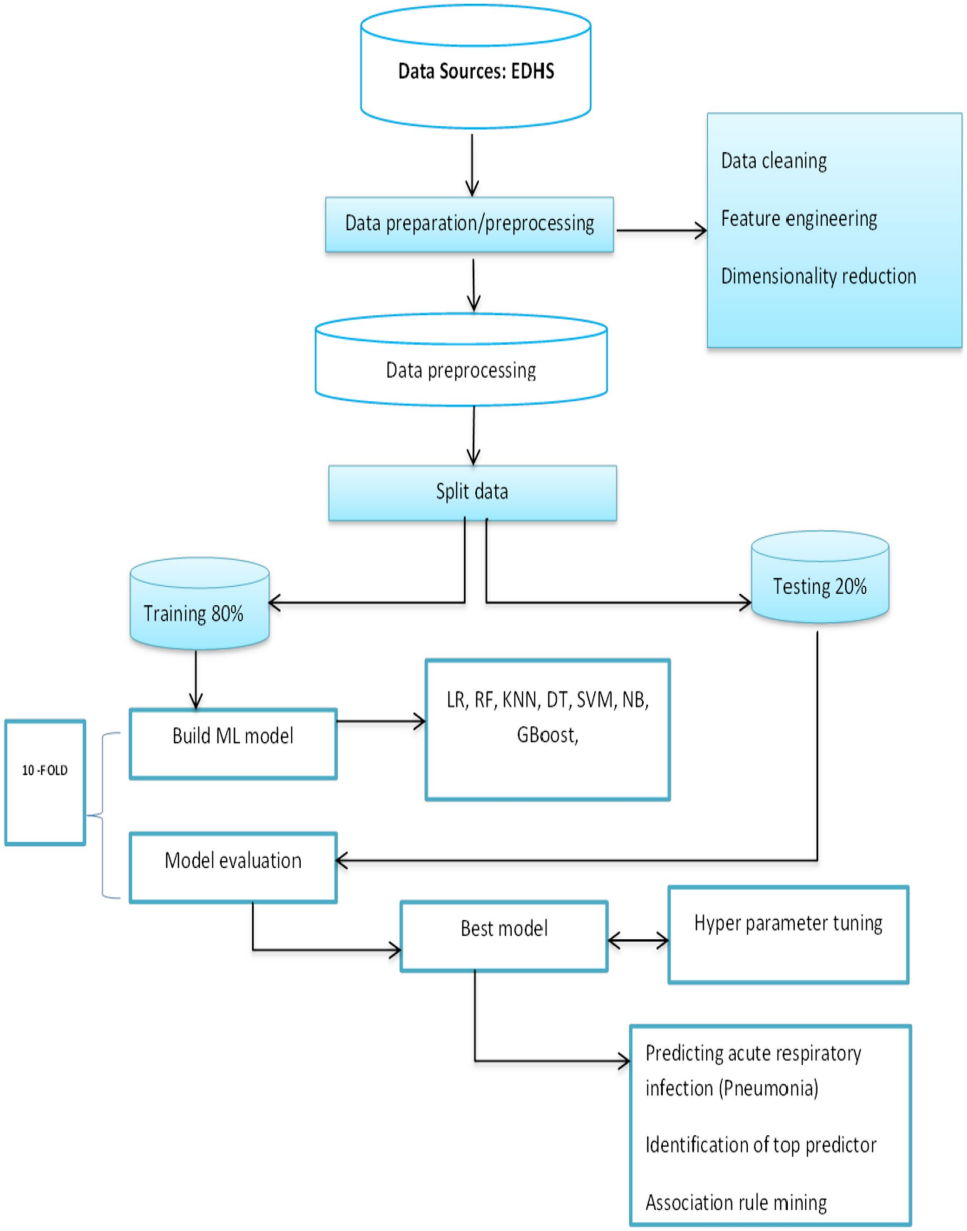
General machine learning process.

All of the earlier tasks are completed at this point in the machine learning process (Figure 2). Prediction is the process of predicting an outcome variable by using independent variables as a base. In this process, key factors found along the way were found to determine diarrhea disease. Among many predictor parameters, the best-performing classifiers with a certain level of accuracy were selected.

### Model interpretation/explanation using Shapley additive exPlanations (SHAP)

In machine learning research, explanations and interpretations of powerful models (usually tree-based models) are rarely found due to their “black box” nature. To minimize the limitations in interpreting machine learning results, we applied a recent SHAP value analysis method, SHAP analysis, based on game theory, which can explain any machine learning model’s prediction, whether globally or locally (18). The fundamental idea of SHAP analysis is to determine each predictor’s marginal contribution to the outcome variable’s prediction result (19, 29).

## Results

### Descriptive results of the background characteristics

Out of the 4,638 study subjects, 1,004 (21.6%) were 48–59 months old, with 35 (3.5%) suffering from diarrhea. The remaining 969 (96.5%) were unaffected by diarrhea, with the majority (50.3%) being male patients. When it comes to the educational status of the respondents’ mothers, approximately 63.4% of the participants were not educated. Approximately 37.4% of the respondents’ families were in the poorest category of wealth (Table 1).

**Table 1:** Socio-demographic characteristics of respondents in the Amhara regional state Ethiopia from 18 January to 27 June 2016 (*N* = 4,638).

				Diarrhea			
				No		Yes	
Variable	Category	Frequency	Column %	Frequency	Row %	Frequency	Row %
Age of child	<6 months/(0)	602	13.0	552	91.7	50	8.3
	6–11 months/(1)	380	8.2	309	81.3	71	18.7
	12–23 months/(2)	842	18.2	708	84.1	134	15.9
	24–35 months/(3)	940	20.3	832	88.5	108	11.5
	36–47 months/(4)	870	18.8	805	92.5	65	7.5
	48–59 months/(5)	1,004	21.6	969	96.5	35	3.5
Sex of child	Male/(0)	2,335	50.3	2080	89.1	255	10.9
	Female/(1)	2,303	49.7	2095	91.0	208	9.0
Type of place of residence	Urban/(1)	883	19.0	790	89.5	93	10.5
	Rural/(2)	3,755	81.0	3,385	90.1	370	9.9
Highest educational level	No/(0)	2,941	63.4	2,670	90.8	271	9.2
	Primary/(1)	1,118	24.1	986	88.2	132	11.8
	Secondary/(2)	362	7.8	323	89.2	39	10.8
	Higher/(53)	217	4.7	196	90.3	21	9.7
Wealth index combined	Poorest/(0)	1736	37.4	1,594	91.8	142	8.2
	Poorer/(1)	750	16.2	670	89.3	80	10.7
	Middle/(2)	616	13.3	541	87.8	75	12.2
	Richer/(3)	558	12.0	493	88.4	65	11.6
	Richest/(4)	978	21.1	877	89.7	101	10.3
Living children	1–3/(0)	2,408	51.9	2,150	89.3	258	10.7
	4–6/(1)	1,569	33.8	1,410	89.9	159	10.1
	Above 6/(2)	661	14.3	615	93.0	46	7.0
Occupation	Not working/(0)	2,741	59.1	2,471	90.1	270	9.9
	working/(1)	1897	40.9	1704	89.8	193	10.2

### Environmental characteristics of respondents

In this survey, 3,613 (77.9%) used wood fuel as cooking material, of which 365 (10.1%) were affected by diarrheal diseases and the remaining 3,248 (89.9%) were not. The majority, 2,559 (55.2%) of the respondents, had an unimproved water source for drinking and cooking. Most participants in this study [2,937 (63.3%)] did not have media access (Table 2).

**Table 2:** Environmental characteristics of the respondents in the Amhara regional state of Ethiopia from 18 January to 27 June 2016 (*N* = 4,638).

				Diarrhea			
				No		Yes	
Variable	Category	Frequency	Column %	Frequency	Row %	Frequency	Row %
Fuel type	Electricity/(0)	285	6.1	266	93.3	19	6.7
	Charcoal/(1)	437	9.4	389	89.0	48	11.0
	Wood/(2)	3,613	77.9	3,248	89.9	365	10.1
	Others/(3)	303	6.5	272	89.8	31	10.2
Toilet	Improved/(0)	285	6.1	266	93.3	19	6.7
	Not improved/(1)	437	9.4	389	89.0	48	11.0
Source of drinking water	Not improved/(0)	2,559	55.2	2,308	90.2	251	9.8
	Improved/(1)	2079	44.8	1867	89.8	212	10.2
Media exposure	No/(0)	2,937	63.3	1,590	89.6	185	10.4
	Yes/(1)	1701	36.7	2,657	90.5	280	9.5
Stools disposal when not using toilet	Not safe/(0)	4,151	89.5	1817	90.7	186	9.3
	Safe/(1)	487	10.5	3,739	90.1	412	9.9

### Nutritional and co-morbid characteristics among children under the age of 5

Of the total number of participants, 2,726 (58.8%) children did not have stunting, of which 280 (10.3%) were affected by diarrheal disease. The majority of children, 4,094 (88.3%), did not receive any medication for intestinal parasites in the last 6 months. Regarding nutritional status, 1,912 (41.2%) were stunted and 431 (9.3%) were wasted and 5,326 (56.0%) did not receive vitamin A supplementation during this time (Table 3).

**Table 3:** Nutritional and co-morbid characteristics of diarrhea among children the under the age of 5 in the Amhara regional state, Ethiopia from 18 January to 27 June 2016 (*N* = 4,638).

				Diarrhea			
				No		Yes	
Variable	Category	Frequency	Column %	Frequency	Row %	Frequency	Row %
Stunting	Normal/(0)	2,726	58.8	2,446	89.7	280	10.3
	severe/(1)	1912	41.2	1729	90.4	183	9.6
Intestinal parasites drug	No/(0)	4,094	88.3	3,696	90.3	398	9.7
	Yes/(1)	544	11.7	479	88.1	65	11.9
Duration of breastfeeding	Ever /(0)	4,448	95.9	3,999	89.9	449	10.1
	Never/(1)	190	4.1	176	92.6	14	7.4
Wasting	Normal /(0)	4,207	90.7	3,799	90.3	408	9.7
	Wasting/(1)	431	9.3	376	87.2	55	12.8
Vitamin A supplement	No/(0)	2,635	56.8	0	0.0	0	0.0
	Yes/(1)	2003	43.2	2,358	89.5	277	10.5
Rotavirus-Vaccine	Not /(0)	304	6.6	465	90.8	47	9.2
	Vaccinated/(1)	4,334	93.4	270	88.8	34	11.2
Had anemia	No/(0)	2,863	61.7	3,905	90.1	429	9.9
	Yes/(1)	1775	38.3	2,585	90.3	278	9.7
Media exposure	No/(0)	2,937	63.3	1,590	89.6	185	10.4
	Yes/(1)	1701	36.7	2,657	90.5	280	9.5
Had ARI	No/(0)	4,429	95.5	4,042	91.3	387	8.7
	Yes/(1)	209	4.5	133	63.6	76	36.4

### Feature selection

**Figure 3:**
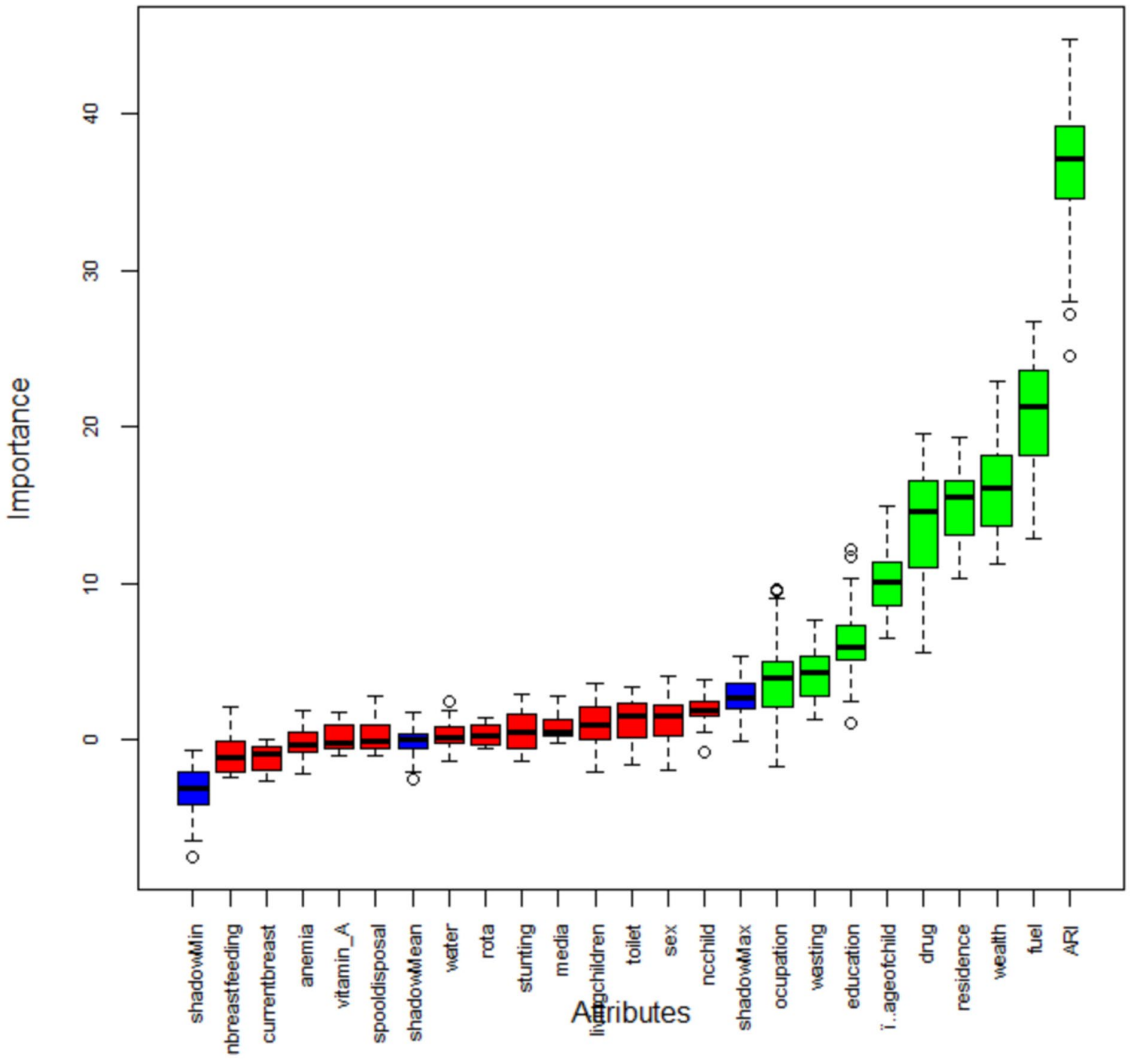
Feature selection using Boruta algorithm. See Appendix for a description of the listed variables.

Feature selection is an important phase of predictive modeling (19, 29). This method is most important when a data set with several variables is provided for model construction. For this study, we used a Boruta algorithm for feature selection, a method commonly used when we want to understand the mechanisms associated with the variable of interest (Figure 2). Using the Boruta feature selection method, 9 out of 22 variables were selected as important features for model construction. ARI, fuel type, wealth, place of residence, and drug were some of the variables that were important for model building and were represented by the blue color. The remaining attributes represented by the red color were rejected by the model because they were attributes unnecessary to the model, such as anemia, wasting, and media exposure (Figure 3).

### Predicting under-5 children’s diarrheal disease status

From the seven models, the random forest algorithm was found to have the highest accuracy of predicting diarrhea with an accuracy value of 81.03%, followed by K-nearest neighborhood (78.46%), decision tree (76.82%), and gradient boosting (75.90%). The positive and negative predictive values for the random forest algorithm were 82.13 and 79.98%, respectively. The sensitivity and specificity for the random forest were 79.64 and 77.19%, respectively. The outcomes of the seven machine learning models, such as decision tree, random forest (RF), naïve Bayes (NB), support vector machine (SVM), K-nearest neighbor (KNN), logistic regression (LR), and gradient boosting (GB) models are presented in Table 4.

**Table 4:** Metrics of model accuracy for each classifier machine learning model as assessed using the test data.

	Machine learning algorithms						
	Decision tree	Random forest	Naïve Bayes	Logistic regression	KNN	SVM	Gradient boosting
	%	%	%	%	%	%	%
Accuracy	76.82	81.03	68.82	66.44	78.46	74.07	75.90
Sensitivity	77.94	79.64	51.15	56.97	79.27	72.73	76.73
Specificity	75.68	82.43	86.73	76.04	77.64	75.43	75.06
PP value	76.46	82.13	79.62	70.68	78.23	75.00	75.72
NP value	77.19	79.98	63.66	63.55	78.70	73.18	76.09
AUC	76.80	86.50	73.70	70.90	78.80	79.50	79.90

AUC, area under the curve; KNN, K-nearest neighbor; SVM, support vector machine; PP, positive predictive; NP, negative predictive.

### ROC curve for the tested models

**Figure 4:**
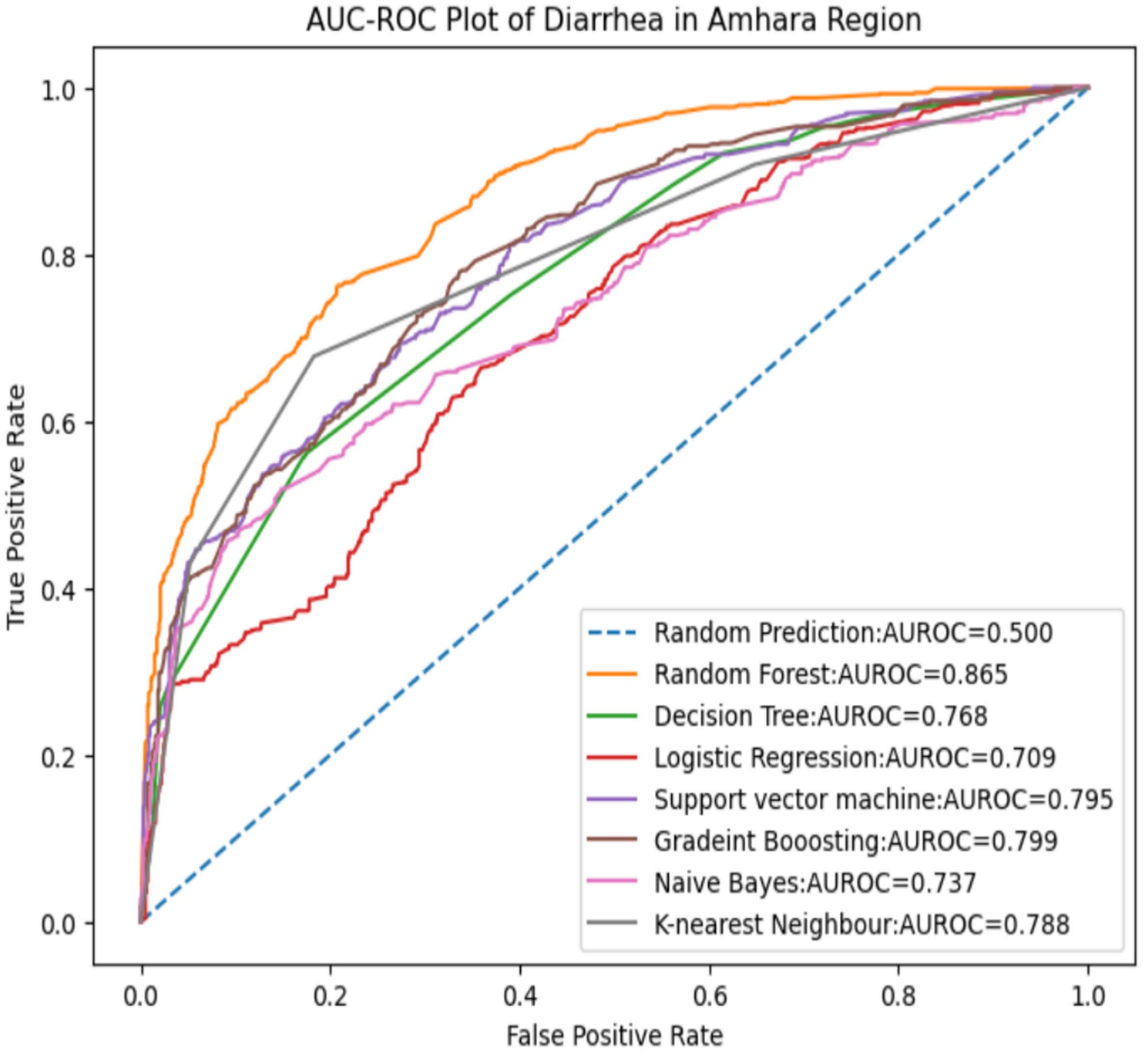
ROC curve for the seven models.

Figure 4 displays a visual representation of the receiver operating characteristics (ROC) curve. The RF model’s curve has the highest AUC value among the seven machine learning models used in this study. AUC is the most effective and strongest model performance measurement—stronger than others such as Accuracy, specificity, sensitivity, positive predictive value, and negative predictive value (Table 4)—in differentiating between children who have diarrhea and those who do not.

**Figure 5:**
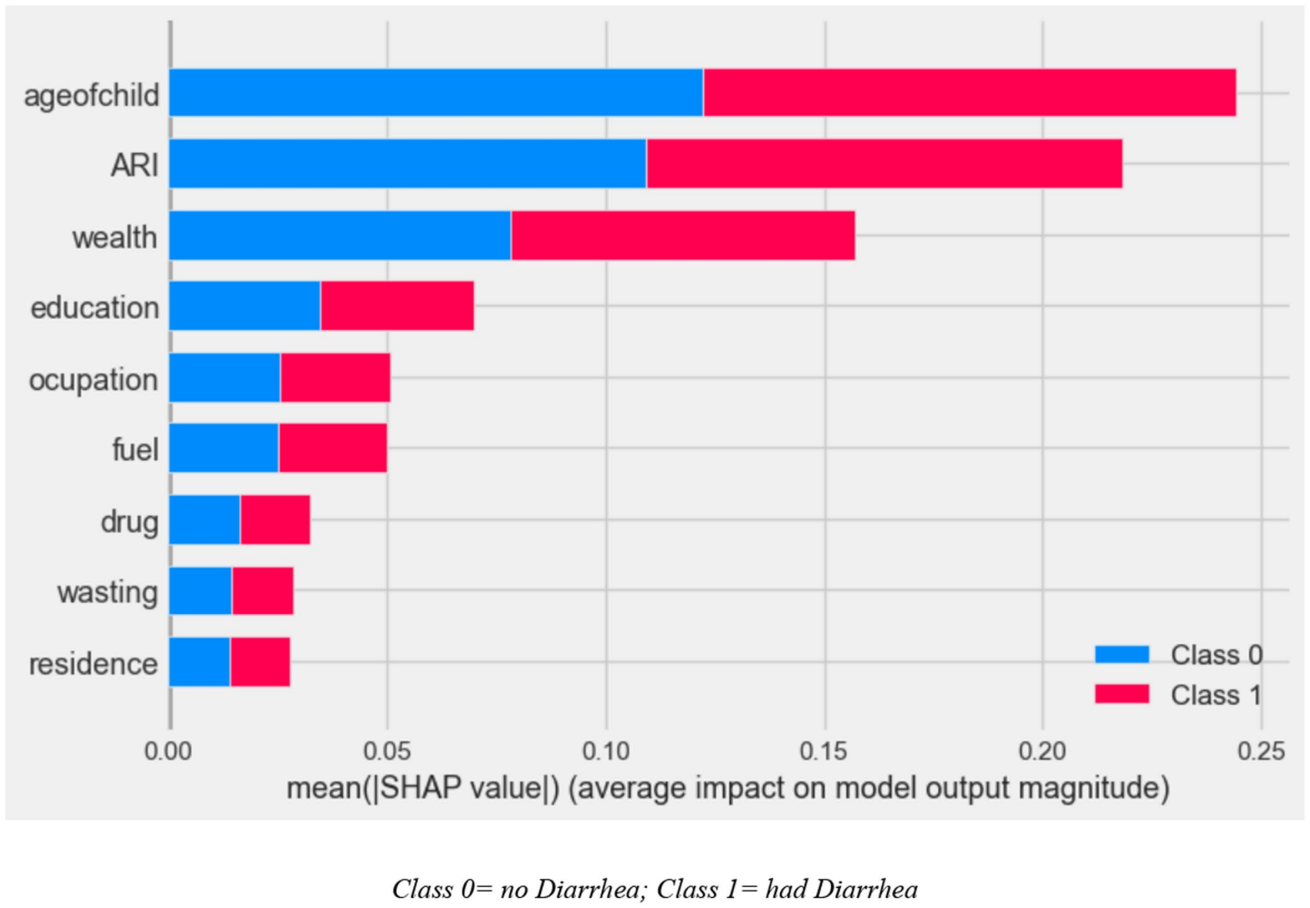
SHAP global importance plot of optimized random forest model.

Based on this result, the next step is to determine the magnitude of the predictor variable using random forest model-based SHAP value, as shown in Figure 5.

The SHAP global importance scores for the top nine factors using the optimized random forest model are shown in Figure 5. The global feature’s contribution toward the predicted diarrhea is also displayed. Higher mean absolute SHAP values indicate a greater influence from the predictors, which are arranged in descending order of their impact on the outcome variable prediction. The results revealed that the most important factors to predict diarrhea are child age, ARIs, wealth, mother’s educational status, mother’s occupation, types of cooking fuel in the household, intestinal parasite drugs, children who had wasting, and types of residence (Figure 5).

#### Model interpretation and justification

**Figure 6:**
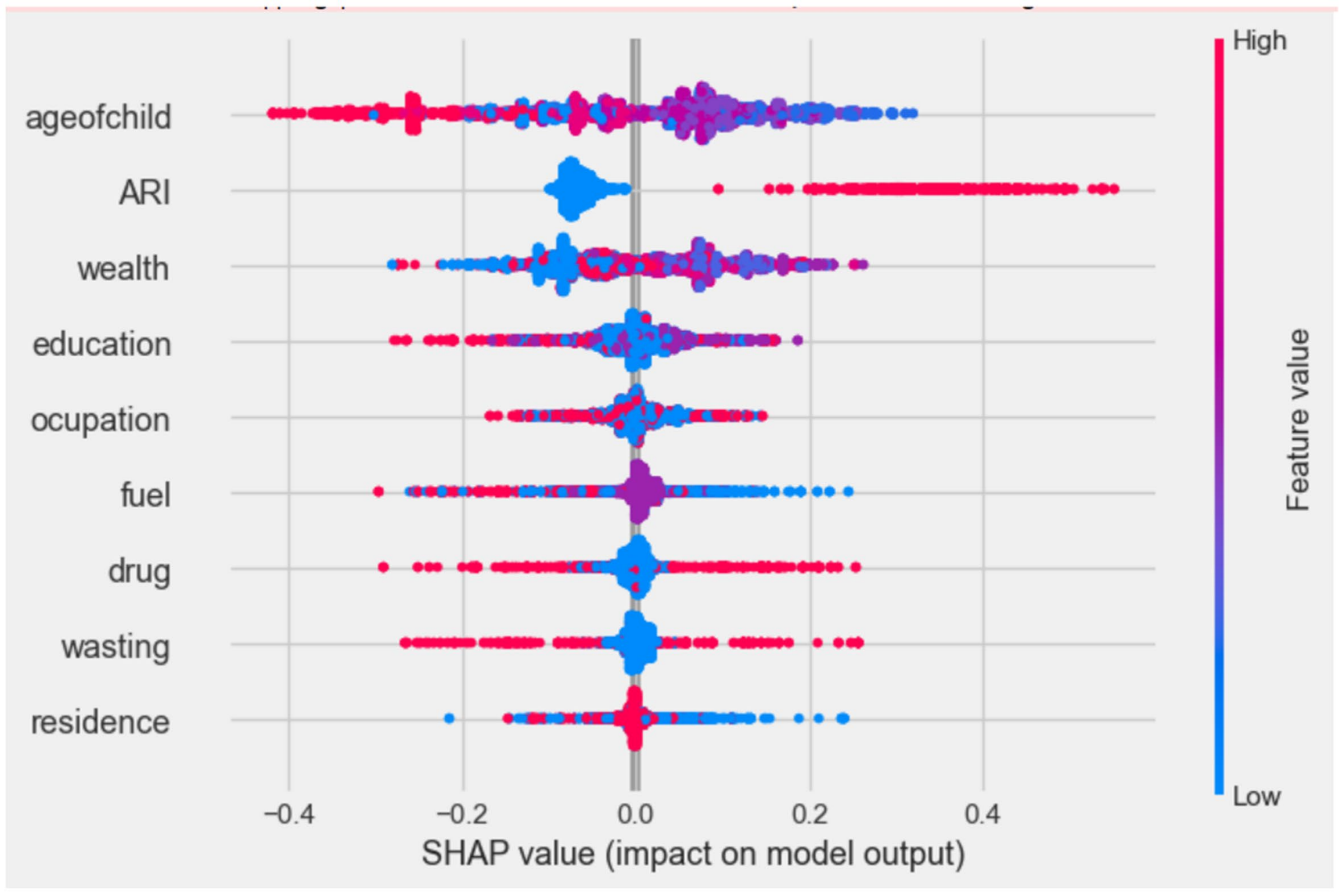
Beeswarm plot, ranked by mean absolute SHAP value generated by optimized random forest model.

To give a comprehensive picture of how the variables affect the model’s predictions across the board, beeswarm plots were used. Figure 5 shows the distribution of each predictor’s effects on the output of the model (i.e., diarrhea prediction) by graphing each sample’s Shapley value for that specific predictor. The significance and correlation between each of the top nine features on the outcome variable are shown by the points on this beeswarm plot, which represent the Shapley values of the features linked to diarrheal disease. The higher and lower values of each predictor’s variable are represented by red and blue in the figure. The probability of diarrhea is higher at points that are in line with the red and lower (protective) values represented in blue (Figure 6).

**Figure 7:**
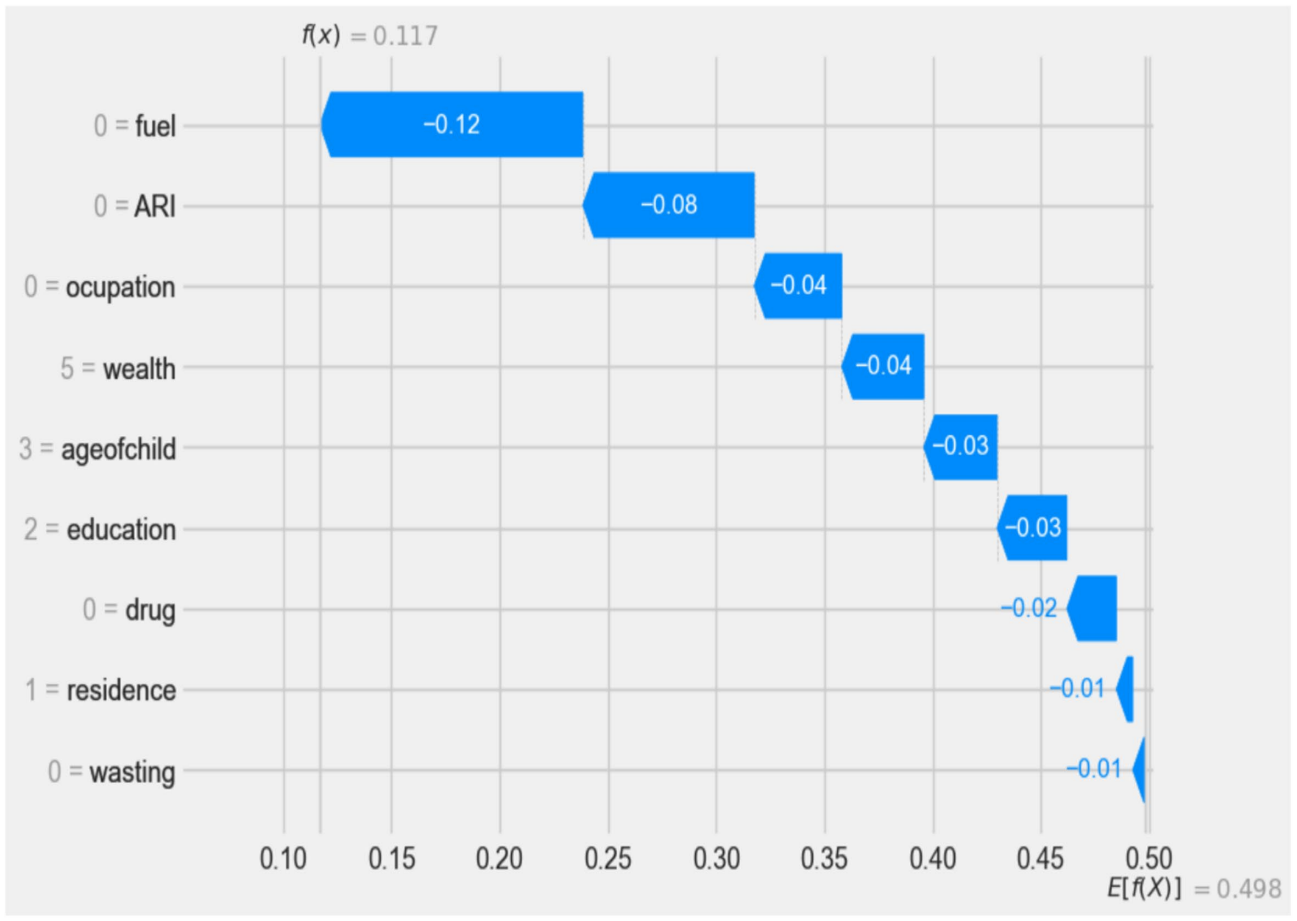
Waterfall plot displaying prediction of the diarrhea positive observation.

Waterfall plots were utilized to explain the model prediction about the diarrhea-positive observations. The waterfall plots in Figure 6 start with the expected value of the model output on the x-axis (E[f(X)] = 0.5), which is the initial prediction for the sample before taking feature contributions into account. Usually, this baseline prediction represents the dataset’s average or most frequent prediction. If the model output for a given observation is greater than this value (E[f(X)]), it indicates a positive class (i.e., diarrhea positive), while results below this threshold indicate that there is “No Diarrhea” in the negative class. As a result, for the first observation, the expected value output is moved to the final model output (f(x) = 0.117), which is categorized as a positive class (had diarrhea) by combining the positive (in red) and negative (protective) contributions (in blue), and it is also used to identify local or individual predictability of the feature (Figure 7).

According to the waterfall result, electricity as a cooking material in the family(0 = fuel), being a child without ARI history (0 = ARI), mothers who did not work (0 = occupation), families with the highest wealth status (5 = wealth), age of child less than 6 months (3 = age of child), secondary educated mother (2 = education), no intestinal parasites drug history (0 = drug), living in an urban area (1 = residence), and children who had not experienced wasting (0 = wasting) have a low impact (protective) on diarrhea prediction (indicated by blue), respectively.

## Discussion

This study provides a brief overview of the prediction of diarrheal disease and its determinants in children under the age of 5 in the Amhara Regional State, Ethiopia, using machine learning techniques. Algorithms tested include random forest, decision tree, naive Bayes, K-nearest neighbors (KNNs), support vector machine (SVM), logistic regression, and gradient boosting. Among these algorithms, the random forest model had the highest prediction accuracy and AUC statistics, indicating its superior prediction ability compared to the other models used in this study. By conducting an ex-additive SHAP value analysis of the best-performing algorithms (random forest), the researchers identified the key risk factors associated with diarrhea.

This study showed that the type of cooking fuel used in the children’s family was significantly associated with the diarrheal disease among children under the age of 5 in the Amhara region of Ethiopia. Family members or caregivers of children who used electricity to cook food are more caring (log odds of −0.12) than their counterparts. This could be due to the susceptibility of an unhygienic food preparation environment to food contamination by various bacteria. This finding is supported by study results from 217 DHS program surveys, which show that diarrheal disease is more influenced by sanitation than water conditions (30).

This study found that diarrhea had a significant impact on young children under the age of 5, particularly those with a history of ARIs. Interestingly, children with no history of ARI were found to have a protective effect against diarrhea compared to children with a history of diarrhea, with a log odd of −0.08. This finding is consistent with a study conducted in India, Bangladesh, and developing countries (31–34). The study suggested that this link may be due to reduced immunity in children with co-existing medical conditions such as ARI, making them more susceptible to diseases such as diarrhea.

In this study, a lower risk of diarrhea was observed in children under the age of 5 with the richest wealth index (by the log odd of −0.04) compared to children with lower and middle wealth indexes. This finding is consistent with findings in sub-Saharan Africa, Iraq, and India (34–37). Wealth has a direct impact on access to sanitation and basic water services. Poor households are more likely to use poor sanitation and unimproved water, making children in these circumstances highly vulnerable to infections, such as diarrhea (38). That is also because wealthier families can usually afford to provide better nutrition and medical care for their children. Wealthier households can also reduce their children’s exposure to contaminated water and unsanitary environments. Furthermore, this study showed a significant association between diarrheal disease and child age. In this study, children aged 24 to 35 months were more protective than younger children for diarrheal disease (log odd of −0.03). The results are consistent with previous research conducted in Ethiopia (39), Indonesia (40), Kenya (41), and India (32) which also found that the prevalence of diarrhea in children aged 6 to 11 months was highest. Other results in Myanmar (42) showed that the combined morbidity of diarrhea and ARI was highest in children aged 12 to 23 months, which is also similar to our results. The disease burden was higher in younger age groups. This result indicates the association between diarrhea treatment and age, implying that diarrhea treatment increases with age. The possible reason for this is that as children grow older, they develop immunity and are able to interact better with their environment by avoiding unsanitary areas and eating healthily, suggesting that the incidence of childhood illnesses decreases with age (41, 43, 44).

According to this study, the mother’s occupation was associated with diarrheal disease in children under the age of 5. Children whose mothers’ were unemployed are more protected against diarrheal disease (log odd of −0.03) than (45) children whose mothers worked. This result is consistent with other studies in Ethiopia and some sub-Saharan countries (46–48). This could be because mothers who are currently working may not have enough time to care for their children, as they spend most of their time at work to increase family income, while mothers who are not currently working usually have time for the care of their children and can minimize their children’s exposure to contaminated objects (35).

This study revealed that the prevalence of diarrhea was lower among children whose mothers had secondary education and higher (with a log odds of −0.03), compared to their counterparts. This finding is similar to a study conducted in sub-Saharan Africa (35), Ghana (49), Nepal (50), and Brazil (45). Educating women can improve their knowledge, attitudes, and practice of basic preventive measures such as proper breastfeeding, child nutrition, water purification, and healthier child care (51, 52). This shows the importance of improving the content and quality of education (e.g., including health education and promotion in the school curriculum, even at low levels of education). Educated mothers also tend to make informed decisions about preventative measures such as vaccinations, proper nutrition, and breastfeeding, which can strengthen their child’s immune system and reduce the likelihood of diarrheal disease (41).

Although the importance of the variable is less compared to the other variables in this study, the study found that urban residence provided greater protection (log odds of −0.01) than their counterparts. This study is consistent with the study conducted in Bangladesh (53). Children who had taken intestinal parasite drugs were more protective (log odd of −0.02) than their comparison. This is always true because children who take medications for intestinal parasites provide better protection than children who do not take medications for intestinal parasites. Finally, this study showed that children who were not affected by wasting showed a stronger protective effect (log odd of −0.01) than their comparison subjects. This result is supported by a study (54) which found that children who are stunted, wasted or underweight have almost twice the risk of developing the bacterium Shigella, the main symptom of which is diarrhea, compared to well-nourished children. A study conducted in the USA also confirmed that thinness and underweight were significantly associated with diarrhea (55, 56). This is due to the direct link between malnutrition and the development of children’s immune systems, which play a large role in disease protection.

### Sample association rules

*Rule 1* (Lift = 1.9): If the child’s age is less than 24 months, the mother’s education level is below the higher education level, the children live in the city, children who have had a history of wasting, and mothers of the child, who were their own job, THEN the probability that the child is affected by diarrhea increases to 96.90%.

*Rule 2* (Lift = 1.9): If the child is 6 to 23 months old, the educational level of the mothers is uneducated, the children taking medication for intestinal parasites, the child has a history of wasting, the mother of child is self-employed, and the child has no history of ARIs, child has a 94.40% likely to suffer from diarrhea.

*Rule 3* (Lift = 1.9): If the child is between 6 and 23 months old, the mother’s education level is primary school age and above, the children’s family wealth status is worst or poorer, the children have a history of wasting, and the mothers of the child do not have their own jobs, THEN the probability that 92.30% of all children will suffer from diarrhea.

### Strengths and limitations

The purpose of this study was to identify factors associated with diarrhea, representing a significant advancement in artificial intelligence. Consequently, the study enhances our understanding of how machine learning techniques can be applied to social science and population health research. Additionally, by advancing knowledge of the causes and risk factors of diarrhea in both rural and urban Amhara settings, the study helps to identify vulnerable populations. Finally, interpretation issues arise from the application of machine learning. Because important variables are chosen by extrapolating patterns from the labeled training data, it can be challenging to interpret the causal effect (57). In particular, interpreting the causal effect can be challenging because the selection of important variables is based on the extrapolation of patterns found in the labeled training data (58). However, we utilize the SHAP score to clarify how the top variables identified are connected to the study outcomes in light of the existing literature.

## Conclusion

This study used machine learning algorithms to develop a predictive model for diarrheal disease in children under the age of 5 in the Amhara Regional State, Ethiopia. By using design science methods, a proposed model was built using various homogeneous ensemble machine learning methods, including random forest, decision tree, naive Bayes, KNN, SVM, gradient boosting, and logistic regression. Nine experiments were conducted, and the random forest algorithm showed the highest performance, achieving an accuracy of 81.03%, sensitivity of 79.64%, specificity of 82.43%, positive predictive value of 82.13%, and a negative predictive value of 79.98%. Depending on this, the researcher recommends developing an AI application to predict diarrheal diseases using a random forest-based algorithm. The study found that mother’s wealth index, mother’s occupation, mother’s education level, type of residence, children’s age, intestinal parasite medications, and he type of fuel used for cooking was significantly correlated with diarrhea in this population. Furthermore, the study highlighted that children without ARIs and children who had no history of wasting are crucial factors in improving child health outcomes in the Ethiopia’s Amhara Regional State. This result implies that ML models may uncover previously unknown insights or generate various variables that could be crucial for informed policy-making that have not been captured by classical methods. This information can be valuable for policymakers in developing effective strategies to combat diarrheal disease in this population. We therefore recommend that the implementation of programs aimed at reducing diarrhea in children under the age of 5 living in the Amhara region should focus on addressing socioeconomic barriers that limit mothers’ access to wealth, working environment, type of fuel for cooking food, and education as well as children’s nutrition and health access.

## Data availability statement

The original contributions presented in the study are included in the article/Supplementary material, further inquiries can be directed to the corresponding author.

## Ethics statement

The studies involving humans were approved by the researchers received the survey data approval letter from the USAID DHS program after registering with the link https://www.dhsprogram.com/data/dataset_admin/login_main.cfm and then the researchers of this study maintained the confidentiality and privacy of the data. The studies were conducted in accordance with the local legislation and institutional requirements. Written informed consent for participation in this study was provided by the participants’ legal guardians/next of kin.

## Author contributions

AK: Conceptualization, Data curation, Formal analysis, Funding acquisition, Investigation, Methodology, Project administration, Resources, Software, Supervision, Validation, Visualization, Writing – original draft, Writing – review & editing. AA: Writing – original draft, Writing – review & editing. EA: Writing – original draft, Writing – review & editing. AY: Writing – original draft, Writing – review & editing, Supervision, Visualization.

## Appendix 1

**Table:**

Code of variable	Description
Ageofchild	Age of children
Livewith	Child lives with whom
Vitamin_A	Vitamin A Supplement
Sex	Sex of child
Drug	Drug for intestinal parasites
Wasting	Wasting
Stunting	Stunting
Breastfeeding	Duration of breast feeding
Anemia	Had anemia history
Water	Source of drinking water
Curentbreast	Currently breastfeeding
ARI	Antiretroviral infection
Livingchildren	Number of living children
Occupation	Mothers’ occupation
Toilet	Type of toilet facility
Rota	Received Rotavirus 1 vaccine
Education	Mothers educational level
Nfamember	Number of family member
Fuel	Type of cooking fuel
Wealth	Mothers’ wealth index
Residence	Type of residence
Spooldisposal	Disposal of youngest child’s stools when not using toilet
Media	Media exposure
Distance	Distance to health facility
Ncchild	Number of living children
